# The cultural evolution of fertility decline

**DOI:** 10.1098/rstb.2015.0152

**Published:** 2016-04-19

**Authors:** Heidi Colleran

**Affiliations:** Institute for Advanced Study in Toulouse, 21 allee de Brienne, Toulouse 30151, France

**Keywords:** fertility decline, cultural evolution, demographic transition

## Abstract

Cultural evolutionists have long been interested in the problem of why fertility declines as populations develop. By outlining plausible mechanistic links between individual decision-making, information flow in populations and competition between groups, models of cultural evolution offer a novel and powerful approach for integrating multiple levels of explanation of fertility transitions. However, only a modest number of models have been published. Their assumptions often differ from those in other evolutionary approaches to social behaviour, but their empirical predictions are often similar. Here I offer the first overview of cultural evolutionary research on demographic transition, critically compare it with approaches taken by other evolutionary researchers, identify gaps and overlaps, and highlight parallel debates in demography. I suggest that researchers divide their labour between three distinct phases of fertility decline—the origin, spread and maintenance of low fertility—each of which may be driven by different causal processes, at different scales, requiring different theoretical and empirical tools. A comparative, multi-level and mechanistic framework is essential for elucidating both the evolved aspects of our psychology that govern reproductive decision-making, and the social, ecological and cultural contingencies that precipitate and sustain fertility decline.

## 1. Introduction

The global transition to low fertility is one of the most striking cultural convergences in human history. Over the past 200 years, people from different religious, linguistic, ethnic and cultural groups, living in economies with different histories and value-systems, are increasingly limiting their families to around two or fewer children. How has this social norm evolved? Can it last? And can we connect theories about how people make personal reproductive decisions with the evolution of a broader culture that increasingly values and rewards smaller families?

For decades, researchers of demographic transition have argued that economic, cultural, ideational and sociological factors are too deeply intertwined in this process to be completely isolated from each other [1–5]. Asserting the causal primacy of one of these dimensions is therefore problematic. Demographic transitions are also multi-level phenomena, driven by nested sets of social interactions [6]. These involve people living in social networks, kinship groups, socioeconomic classes and communities, followed by interactions between these entities and between regions and countries in a global network. The challenge then is to explain both substantial within- and between-society variation in the trajectories of fertility decline, and the global convergence on low fertility as a general process, while doing justice to the coevolutionary nature of economic, cultural and population change.

Evolutionary theorists have produced many abstract treatments of the dynamics of fertility decline at different levels of analysis. Evolutionary anthropologists have been testing aspects of these models for at least 30 years [5,7–10]. This work has mostly focused on the predictions of optimality approaches, or on critically comparing how socioeconomic and sociocultural characteristics of individuals predict their fertility outcomes [11–13]. There are now strong lines of enquiry on reproductive competition and cooperation within families, reproductive trade-offs and other allocation decisions, life-history and context-dependent variation in reproductive behaviour [14]. Much of this is consistent with mainstream demography but with additional insights gained from thinking about why any animal would ever reduce fertility in a time of plenty.

In principle, causal models combining economic and cultural factors are a shared objective. Empirically, this is a daunting task. In practice, little work has been done to actually synthesize different conceptual approaches [12,15,16], with little explicit focus on how cultural transmission contributes to—or derails—adaptive reproductive behaviour. Partly, this is because many evolutionary anthropologists and demographers identify as human behavioural ecologists (HBEs), taking inspiration from animal models of behaviour in evolutionary biology and sharing common conceptual ground with economists [17]. They share interests in life-history theory, resource-allocation strategies, context-dependent adaptation and fitness optimization [18,19]. However, it is also partly because theoretical models of cultural evolution (CE) have been less widely read and are thought more difficult to test.

Cultural evolutionists have long been interested in the demographic transition. Founding texts in the field [20,21] emphasized fertility decline as a canonical example of how cultural transmission can drive behavioural outcomes that do not maximize genetic fitness. CEs regularly raise fertility decline as a counterpoint, both to exclusive reliance on optimality approaches and to assumptions about ‘adaptive lag’ in contemporary human behaviour [22,23]. Given that low fertility rates in the advanced economies of the world cannot be considered fitness-maximizing [24–27], CE is gaining traction among empirical researchers [12,13,15,16,28,29]. But only a few models are widely cited and discussed [8,14].

Moreover, some conceptual overlaps between CE and HBE can make them difficult to distinguish as alternative explanatory frameworks. Perhaps as a result, CE theory has not made inroads into mainstream demographic thinking, despite a growing representation of evolutionary research in that literature [30]. This is unfortunate, given demographers' longstanding debates about demand versus ideation theories of demographic transition [1,31,32], their interest in the relative contributions of cultural and economic processes to this phenomenon [32–34], and their aspirations to integrate micro- and macro-level understandings of demographic change [6]. Indeed, the main cleavages in the evolutionary literature closely parallel those in demography, with debate surrounding the role of rational-actors [28,35] and methodological individualism, dichotomies between economic and cultural explanations of fertility decline [12,28] and the importance of social versus individual learning [21,35,36] (though defined differently than in demography [37], see §2b).

Evolutionary research on fertility decline needs to address the multi-level nature of human social interaction, which generates opportunities for evolutionary dynamics that both involve reciprocal causation and are not easily reduced to individual characteristics [15,23,38]. A multi-level approach highlights that different parts of the ‘system’ of demographic transition might be driven by different evolutionary processes. Fertility decline has three distinguishable phases—the origins, spread and maintenance of low fertility—each of which may require different theoretical and empirical tools and may occur on different scales. Rather than pitting particular frameworks against each other, developing and testing hypotheses that draw on a principled synthesis of theoretical outlooks will undoubtedly be more productive.

We need clearly testable predictions from CE models and clarification of conceptual overlaps that are hampering theoretical integration. HBE's commitment to the behavioural gambit [39]—a black-box approach to how optimization problems are solved—allows researchers to focus on the fitness costs and benefits to reproduction and on the evolutionary functions of reproductive decision-making [18]. But the causal structure linking individual decisions to those of other people and to higher-level patterns remains poorly understood. Mechanistic explanations of fertility decline are needed at the very least because there are multiple ways for individuals and populations to reach (or to miss) optimal solutions [39–41].

This review argues for a deeper integration of CE theory into (evolutionary) demography, as a means to develop multi-level models of fertility decline that emphasize the coevolution of economic and cultural change and not the *a priori* privileging of one over the other. I begin by reviewing some basic conceptual differences between different evolutionary approaches, noting similarities and departures from standard demographic thinking, before briefly outlining the CE work published to date. This is followed by conceptual overlaps and a critical comparison of how data on demographic transitions are interpreted by different sub-fields. Finally, I offer some new directions that can generate novel hypotheses about the dynamics of fertility decline from an evolutionary perspective.

## 2. Basic differences affecting how low fertility is interpreted

There are a number of different ways to think about fertility decline from an evolutionary perspective (table 1). Each raises challenging and unresolved theoretical questions about: the nature of reproductive success in the contemporary world, the psychological mechanisms we think are at play, whether low fertility should be considered adaptive or not, what level of analysis is necessary and what aspects of contemporary and recent environments precipitate or slow down reproductive change.

**Table 1.** A comparative overview of evolutionary hypotheses on the fertility transition. Theoretical approaches are organized into three different phases: the origins, spread and maintenance of low fertility.

	Evolutionary Hypothesis	Low fertility adaptive?	Level of analysis	Psychological mechanisms	Causal model	What's different about market economies?	Key publications
ORIGINS	Adaptive lag	no	individual	Natural selection has not shaped psychologies to optimize family size	Sex and reproduction are decoupled; selection pressure low	Modern contraception	Laland & Brown [22]
	Embodied capital maximisation^a^	no	individual	Parents invest in skills and knowledge acquisition	Energetics of reproduction decoupled from resource and skill accumulation; QQTO	Wage-labour markets require embodied capital, investment potentially unlimited	Kaplan [10]; Kaplan *et al.* [24]; Grafen [42]
	Coevolution of wealth inheritance and fertility^a^	no	individual	Parents invest in wealth or status accumulation	Wealth inheritance affects ability to marry and reproduce; QQTO	Unlimited opportunities for wealth creation, status competition	Mace [7,43,44]
	Stabilizing-/lineage- selection on long-term reproductive success	yes	lineage	Status striving; avoid lineage extinction	Intermediate clutch sizes maximize fitness of next generation or avoid lineage extinction	Social stratification; wealth inheritance; competition between lineages	Low *et al.* [45]; Mueller [46]; Kaplan *et al.* [24]; Hill & Kern-Reeve [47]; Boone & Kessler [48]
	Social mobility strategy	yes	family	Parents invest in wealth or status accumulation	Differential marginal reproductive returns to up- or downward mobility	Social stratification; Reproductive failure at the bottom of the hierarchy	Rogers [49]; Harpending & Rogers [50]; Rogers [51]
	Variance or risk compensation	yes	individual	Parents invest in wealth or status accumulation	Over-reproduce when uncertainty is high, under-reproduce when low	Mortality decline	Winterhalder & Leslie [52]; Leslie & Winterhalder [53]; Low *et al.*[54]
	Cultural versus biological 'parentage'	no	individual	Individuals invest in own wealth or status accumulation	Reduce fertility to maintain or obtain prestige position	Stiff competition for prestige-positions	Richerson & Boyd [55]; Boyd & Richerson [21]
	Loss of kin influence	no	individual (or group)	Teaching bias; give pronatal advice to kin	Loss of kin in social networks reduces their potential impact on reproductive norms	Social networks widen and become more diffuse	Newson *et al.* [56,57]
SPREAD	Cultural niche construction	no	group	Frequency-dependent bias; Conformity bias	Distribution of cultural trait 1 alters percolation of cultural trait 2 via horizontal/oblique transmission	Mass education, communication networks, social networks widen	Cavalli-Sforza & Feldman [20]; Ihara & Feldman [58]; Kendal *et al.* [59]; Borenstein *et al.* [60]; Fogarty *et al.* [61]
	Prestige- / success- biased transmission	no	individual	Prestige- or success-biased copying	One-to-many transmission, small 'effective' population size of traits; cultural 'drift'	High status people have sacrificed fertility to keep their prestige-positions	Richerson & Boyd [35]; Henrich & Gil-White [62]
	Cultural group selection	individual no; group yes	group	Frequency-dependent bias; Conformity bias; Selective migration; Prestige-bias	Competition between groups for development, resources, immigrants	Globally interconnected networks of mutual investment and competition	Richerson *et al.* [63]; Richerson & Boyd [64]; Dang [65]; Dang & Bauch [66]; Henrich [67]
MAINTENANCE	Cultural evolutionary population dynamics	no	group	Cultural innovation; vertical and horizontal transmission of preferences and lifestyles; frequency-dependent bias	Lifestyle innovation is faster than natural selection on low fertility	Lifestyle innovation is faster, mass communication	Kolk *et al.* [68]; Ghirlanda & Enquist [69]; Ghirlanda *et al.* [70]

^a^These hypotheses also make partial use of the logic of adaptive lag, in that they argue the strategy was adaptive in the past, even though the reproductive outcomes today may not maximise fitness (QQTO: quality–quantity trade-off).

Broadly speaking, there are three main approaches. First, we could decide that fertility decline is a mismatch (a case of adaptive lag) [22]. Our psychologies did not evolve to explicitly focus on family size but to strive for sex and status. Two hundred years is not enough time for us to adapt to the abundant resources of today's market economies and efficient contraceptives can now sever the link between sex and reproduction. Many evolutionary psychologists take this approach, but there is almost no theoretical work on fertility decline from this perspective, so I will not discuss it further. Second, we could assume that our psychologies evolved to do a good job of parsing the costs and benefits of reproduction in any environment. We would then focus on the trade-offs imposed by contemporary environments, how these might differ from ancestral ones, and how they might incentivize low fertility [71]. This route is taken by many HBEs. Third, we could assume that reproduction, like all behaviour, takes place within a cultural environment that itself defines opportunities and payoffs and the way these are perceived, through shared norms and values, themselves the product of CE in structured populations. Here we might on one level want to understand how biases in the way we learn from one another—biases that evolved to help us efficiently acquire adaptive behaviour—affect reproduction and are subject to new structural, cultural and transmission constraints, or to the lifting of old ones. Or on how variation, competition and selection at multiple levels of social organization conspire to generate downward pressure on fertility. CEs typically take this tack.

### (a) The view from human behavioural ecology

HBE is generally concerned with uncovering and understanding the costs and benefits of low fertility for individuals or lineages by examining constraints on fitness maximization, in particular how fundamental trade-offs, parental investment strategies and reproductive competition drive fertility outcomes. Personal decision-making on this approach is analogous to economic decision-making and research on fertility decline often draws on economic models of the family [17] and extensions and variations by evolutionary researchers [10,27,71]. These cost–benefit decisions differ in one important respect: the ‘utility’ being maximized—fitness—is explicit. If behaviour can be considered optimal (i.e. fitness-maximizing, given constraints), HBE is not generally concerned about the decision-mechanisms that generate adaptive fit between behaviour and environment [19]. While recognizing that social interactions and cultural transmission are important—the term ‘socio-ecology’ is employed to reflect this understanding—HBE generally considers these as constraints on (information relevant to) adaptive decision-making, and thus as proximate mechanisms [18]. This stance is changing, because the behaviour in question is clearly not one that maximizes fitness [24–26].

In all species, the energetics of reproduction is tied to resource accumulation, but humans evolved in a skill-intensive foraging niche, which requires investment in ‘embodied’ capital (skill, physical strength, local knowledge) to obtain resources, as well as extra-somatic and other forms of wealth [10]. A leading explanation of fertility decline is that market economies increase the returns to parental investment in new forms of embodied capital (in the form of education), generating trade-offs between the quality and the quantity of children produced [10,72–74]. Subsistence economies that rely on extra-somatic wealth for marriage and reproductive opportunities may also generate conditions for the coevolution of low fertility preferences and social institutions promoting inheritance of material wealth [7,10,43,49,50].

Early models of fertility decline [7,43] showed how increasing the opportunity costs of both marriage and raising children can in principle decrease optimal fertility to very low levels. Additionally, if children differ in their ‘reproductive value’ (i.e. expected reproductive success), then parents should exhibit reproductive restraint, maximizing the cumulative value of their children, and not simply the number [42]. Null or negative relationships between wealth, status and the number of children born, as are typically found in post-demographic transition populations, do not rule out a positive relationship between wealth and reproductive value [26,27,42]. ‘Arms races’ of parental investment in quality could then drive the evolution of low fertility, especially in socially stratified populations where competition may occur within rather than between social strata [42,44,45,47,48].

A number of researchers have proposed that long-term fitness, or the success of lineages, is theoretically increased by decreasing fertility in the short-term (e.g. [49], though see [51]). Diluting resources between lots of children increases the chances that they will be downwardly mobile, so low fertility could be an adaptive strategy to help avoid this outcome (or increase the probability of upward mobility) [50]. If individuals are risk- or variance-sensitive, over-producing when mortality uncertainty is high and under-producing when it is low [52,53], then periodic environmental crises or stochastic fluctuations could also make it sensible to pursue a low fertility strategy, again enabling lineages to survive [48] (see also [45,54,75]). This makes sense because if resources became limited, and access to them was unequal, then wealthy and/or high-status families could thrive at the expense of the rest of the population. That being said, where this kind of scenario has been modelled [45], the conditions under which low-fertility, late-breeding lineages persist at the expense of high-fertility, early-breeding ones have proved extremely difficult to create.

Ultimately, for low fertility to be evolutionarily as opposed to economically advantageous, there has to be a fitness payoff to having fewer high-quality children, in either the short or the long term; we need to see low-fertility families ‘cashing in’ on their advantage in reproductive terms at some point. To date, no evidence has justified this assumption [24–26] and a number of important conceptual issues remain unclear. How many generations should we consider, and why switch from a one- to a multi-generational strategy? Why switch the evolutionary currencies being maximized? Do children in smaller families meaningfully differ in their reproductive value? Other important components of fitness include survival to reproductive age, ability to find a partner and ‘recruitment’ of children into the reproducing population, and natural selection operating on some or all of these components could in principle drive fertility decline.

### (b) The view from cultural evolution

CE work relevant to understanding demographic transition has broadly focused on three different areas: (i) how individual learning biases that evolved to optimize social information transmission generate and maintain different frequencies of cultural traits in populations; (ii) how variation and interaction between groups at different cultural equilibria lead to between-group competition, selection and transmission; and (iii) how different channels (modes) of social transmission affect the dynamics of information flow in populations. CE theory takes inspiration from both population genetic models and social psychology, and offers a multi-level approach to social behaviour, emphasizing that individual strategies for adaptation within groups and competition between groups are often co-occurring [76]. CEs commonly define *culture* as ‘social information transmitted via teaching, learning and imitation’ [20,21], similarly to how ‘ideation’ is construed in demography. More broadly though, CE considers cross-culturally and temporally stable institutions and traditions, including the economy itself, to be socially constructed, coevolving entities, and not as wholly exogenous (extrinsic) constraints on decision-making [23]. Unlike optimality models, in many CE models the fitness of a particular cultural trait is inferred from frequency changes in the population, assuming certain learning-rules, rather than by pre-defined utility functions measured purely at the individual level.

Social learning mechanisms evolved to enhance genetic fitness [20,21,77]. CE models distinguish three general types of learning bias: content-, context- and frequency-dependent [77]. Content bias (also called ‘direct’ bias [21]) is similar to cost–benefit analysis (also called asocial or individual learning). The individual selects a cultural trait based on its perceived intrinsic utility. Under context biases, individuals adopt traits based on the characteristics of the person (or social model) who exhibits it (also called ‘indirect’ bias [21]). This allows learners to avoid the costs of asocial learning using some cue about the suitability of social models, for example success in a particular domain, or deference (prestige) shown by third parties [62]. The cost—that these cues may not be related to the behavioural trait in question—generates trade-offs between rapid social learning and the adoption of maladaptive traits [78]. Frequency-dependent biases use the frequency of a trait or behaviour in a group as a cue to its utility. Individuals may weight these frequencies positively (conformist bias) or negatively (anti-conformist bias), generating nonlinear adoption patterns in the population [21,67]. Alternatively, people may simply randomly copy the most frequent behaviours they observe (neutral frequency-dependence). Individuals are expected to trade-off the costs and benefits to social and asocial learning, leading to conditional learning strategies [79]. Within a population, a balance of information production (asocial learning) and ‘scrounging’ (social learning) is needed for behaviour to remain connected to ecological variation [36,77].

Conformist bias is argued to be especially important in maintaining between-group variation, by increasing differences between groups while maintaining similarities within them [80]. This variation, coupled with competition between groups, is the driver of the cultural group selection model of the evolution of aspects of human behaviour [63]. Conformist bias is similarly argued to underlie the S-shaped adoption curves associated with the diffusion of innovations [67,81] by inhibiting uptake when innovations are rare, and accelerating take-off once a critical threshold of adopters is reached. This is similar to how ‘social influence’ works in demography [37], defined as the social power that interpersonal interactions at various levels have over individual decisions, through basic human tendencies of conflict-avoidance, deference to authority, and the sanctioning ability of powerful individuals or institutions. By contrast, the cost–benefit analysis advocated in HBE is more comparable to ‘social learning’ in demography, defined almost as a Bayesian process whereby a set of subjective beliefs are continuously updated through drawing on both asocial and social information [37].

Cultural traits can be acquired from many other people and modified by individuals within their lifetime. They can also make their bearers more visible as social models. Moreover, since information is transmitted horizontally (peer-to-peer) and obliquely (from non-parents of an older generation) as well as vertically (from parents-to-children) [20], there is ample room for maladaptive traits to quickly spread in a population without relying heavily on particular biases for explanatory power. Oblique and horizontal transmission should be most efficient at diffusing new traits in a population because of their asymmetric (one-to-many) nature. Vertical transmission is analogous to genetic transmission and should thus result in a slower process of cultural change [20]. These can be understood as individual level mechanisms (e.g. you receive information from a person defined as a horizontal or a vertical source) or as group-level properties (i.e. the rate of transmission in a group or the proportion of total information transmitted via a particular channel).

## 3. Cultural evolutionary models of fertility decline

While social learning biases are somewhat integrated into evolutionary discussions of fertility decline in HBE, the almost exclusive focus on individual-level mechanisms has led to a neglect of the second two components of CE research outlined above: group-level dynamics and modes of transmission. CE models of fertility decline have focused more on trade-offs between cultural and biological success [21,55] than on those between quantity and quality (as is typical in HBE), but also on how changing social structures [57,60] and dynamics [58,59] of social transmission affect the diffusion of information about reproduction. This research falls broadly into three areas focusing on the origins, spread and maintenance of fertility decline.

### (a) Origins

There is some debate as to whether CE can successfully explain the origins, as opposed to the spread, and perhaps maintenance of low fertility [8], but in fact some early treatments focused on just this aspect [21,55]. Richerson & Boyd [55] showed that small differences in peoples' tendency to become ‘cultural parents’ as opposed to biological parents could lead to natural selection on fitness-reducing behaviour, with status-competition driving trade-offs between cultural and reproductive success [21]. Only one individual can become a CEO, for example, but most people can become parents. Stiff competition for prestige positions in modernizing populations could generate selective pressure for status-acquisition behaviour that requires a trade-off with fertility, consistent with other causal models in HBE, though the mechanisms may differ (table 1). If achieving status (i.e. becoming a cultural parent) is associated with even marginally more social visibility than being a biological parent, then high-status individuals have privileged leverage to ‘asymmetrically’ transmit their values to other people (i.e. one-to-many). Note that this does not presuppose active transmission, only that social visibility makes one's values and trade-offs more easily observable. Note also that I use ‘low fertility preferences’ as shorthand here: these can refer to any preferences that have low fertility as their downstream outcome. Teachers, for example [35], are privileged oblique and horizontal transmitters of educational values, while also embodying the trait being transmitted. From the cultural-traits-eye view then, the high-education phenotype promotes itself better by being associated with high-social status than with high fertility.

### (b) Spread

The most cited CE arguments on fertility decline emphasize prestige-bias in the spread of low-fertility behaviour [35]. Prestige-bias feeds off the process just described; once culturally successful social models exist, other individuals can adopt their fertility-limiting behaviours. More generally, if this kind of one-to-many transmission is dominant, then the ‘effective’ size of the cultural trait population will be small (i.e. the trait will originate in only one or a small number of individuals), allowing other non-selective processes, such as cultural drift, to become important [20,63].

Changing structures of social interaction may also change how transmission biases scale up to produce low fertility patterns at the- population level. Newson and colleagues [56,57] argue that ‘modernization’ is typified by declines in the frequency of kin interactions, owing to a smaller proportion and influence of kin in social networks. They argue that a ‘teaching bias’ predisposes individuals to communicate slightly more pro-natal information to kin than to non-kin, such that kin communications will tend to maximize inclusive fitness [57]. In theory, a weak teaching bias combined with natural selection can maintain high-fertility norms in a population, if social networks are dense with kin [57]. Economic modernization disrupts these conditions, introducing more interactions with non-kin via migration, education and working environments. Over time, incremental changes in the frequency of kin interactions may allow the emergence of population-level reproductive norms that are less pro-natal.

Such group-level characteristics can feed back to influence individual behaviour. Cultural niche construction models are one way to address these kinds of interactions [23]. These examine how the distribution of one cultural trait, such as a preference for education, can transform both the modes and rates by which secondary cultural traits are spread in the population [58–60]. Different cultural ‘backgrounds’ or niches then determine the evolutionary dynamics of information transmission. Different channels of transmission (i.e. horizontal and oblique versus purely vertical) can become more important depending on whether the trait being transmitted has a ‘life cycle’ that (i) makes it more relevant at different life-history stages or (ii) makes non-parental social models particularly salient in transmitting it [21]. For illustration, assume that women initially inherit reproductive norms from their mothers, via vertical transmission. Some values, like those affecting contraceptive behaviour, will not be important until the woman reaches maturity. At that point, a wider array of social interactions exposes women to the reproductive norms of lateral kin and non-kin in their social environment, increasing the amount of information relevant to reproduction that is obliquely and horizontally transmitted.

Rapid ecological change can exacerbate these differences. In slowly changing contexts, and especially in relatively ‘closed’ social groups, reproductive norms may be less variable and information from different people fairly consistent (i.e. the entire population is at a ‘cultural equilibrium’). Old order Anabaptists and other culturally isolated groups, who often have high fertility even when living within low fertility societies such as the USA, are prime examples [35]. When change is relatively rapid, as during economic modernization, parental information about reproduction may be out of date by the time a women reaches maturity, further enhancing the salience of information from conspecifics, locally prestigious and self-similar individuals [62].

In the first of a series of models, Ihara & Feldman [58] examined how a vertically inherited preference for high education affected the percolation of a low-fertility preference via oblique transmission. They assumed that high average education in a group increases the degree to which traits are transmitted obliquely (e.g. from teachers) compared with vertically (from parents). They found that if parents have a slight bias to transmit high-education preferences to their children—perhaps because they want to invest in children's education, transmit heritable wealth or have high-status themselves—then oblique transmission can hitchhike on this process to spread small family size norms faster than could vertical transmission alone. Kendal *et al*. [59] similarly assumed that high average education accelerates the horizontal transmission of preferences for contraceptive use. They examined how horizontal transmission interacts with conformity to a high-fertility norm and with natural selection pressure against low fertility. Their model shows that low fertility preferences can invade the population even if natural selection favouring high fertility is strong, as long as horizontal transmission is prevalent and conformity bias is low. The higher the frequency of parents preferring to transmit values about education, and thus the higher mean education in the offspring generation, the easier this invasion is, and the lower the amount of horizontal transmission needed to get the process going [59].

Borenstein *et al*. [60] extended this framework to examine how these transmission dynamics play out in a network of subpopulations. They found that individuals living in groups with lower average education could still be influenced by the spread of low-fertility preferences in other parts of the population. Interactions between groups can expose people to low-fertility norms at a lower level of education than would be expected if that group were in isolation. This kind of effect could help explain not only why fertility decline begins in more educated groups first, but also why it spreads to different, neighbouring groups at increasingly lower levels of economic development, especially when they share a linguistic, ethnic or religious affiliation that makes social interaction outside the group more likely [6,60,82]. A general pattern emerging from these models is a time lag between mortality and fertility reduction that is consistent with the empirical record [6] (under the reasonable assumption that education affects individual mortality profiles). These time lags depend critically on how average education affects the rate of cultural transmission; they do not emerge when average education is not allowed to play this role [59].

A few models have begun to tackle how age-specific learning patterns influence both cultural transmission dynamics and the demographic (age) structure of a population [20,61,83]. The idea here is that if learning is age-structured or has a life cycle (as outlined above), there is a greater chance that an individual will encounter a horizontally or obliquely transmitted trait as they age. Fogarty *et al.* [61] showed that if different modes of transmission matter for different age-groups, then low fertility values can simultaneously spread quickly in a population and change the population structure via their effects on reproduction. Their model predicts that where horizontal transmission is constrained, fertility declines driven by the spread of cultural norms will not occur.

### (c) Maintenance

Other models have examined how low fertility could be maintained over the long-run ([68], see also [69,70]). Kolk *et al*. [68] predict that low fertility is unsustainable unless there is a continuously high rate of cultural ‘innovation’ in lifestyles promoting small families. They distinguish between ‘lifestyle preferences' (inherited preferences for low or high fertility) and actual ‘lifestyles’ themselves (i.e. adoption of a high- or low-fertility outcome). Under the assumption that a latent preference for low fertility must exist in part of the population (the initial conditions assume that 80% of the population has this preference), they develop two models to examine how vertically transmitted reproductive preferences fare against a bias to adopt low-fertility behaviours observed in other peers (including parents). The central mechanism is the intergenerational correlation between parents' and children's preferences and outcomes. Their first model predicts that under high-fidelity vertical transmission, high-fertility preferences and behaviours will inevitably dominate the population. This happens through effects on both fertility and intergenerational correlations. As low fertility initially spreads, variation in intergenerational correlations grows, but they become less varied again as high-fertility behaviours take over, i.e. natural selection kicks in. Their second model examines what happens when assumptions about the fidelity of vertical transmission are relaxed and individuals can have multiple preferences influencing fertility. This effectively increases the rate of ‘lifestyle innovation’, dampening the tendency for high-fertility preferences to become dominant, and because individuals can now have preferences that are different from their parents, low-fertility lifestyles can persist [68].

## 4. Conceptual gaps and overlaps

A major roadblock to successfully integrating the CE and HBE frameworks is the fact that different evolutionary models often generate the same empirical predictions at the individual level, making competing hypotheses difficult to identify [8,11], and their different epistemological contributions unclear. The following examples highlight some aspects of this problem.

### (a) Social learning as a proximate mechanism

An influential explanation of demographic transition is that the real or perceived costs of children increase as living standards go up, with the opportunity costs to reproduction becoming disproportionately large for the wealthy and highly educated members of a population [8,10,84]. Individual fertility reductions, under this essentially economic approach, can be interpreted as best responses to investment or information constraints [17], with social learning acting as a proximate, but not an alternative, causal mechanism. Social learning strategies evolved to generate adaptive fit between environment and behaviour; they do not in and of themselves affect the broader reproductive strategy. But it is new information that can lead to behaviour change, not just the fact that we have social learning mechanisms. Cultural norms and values are themselves evolving, and not necessarily in tandem with reproductive success. While humans have been social learners at a large scale for a very long time, some institutional changes like mass communication and education systems have profound effects on the scale and speed of information passing (often horizontally) through populations. Socially, spatially, culturally and demographically structured interactions additionally affect what goes into our decision-making mechanisms. Perceptions about the marginal gains from embodied capital investment and about greater opportunity costs are subject to these constraints. Of course, some opportunity costs may be more fixed than others, trade-offs between time spent on childbearing and education/work being one example. In explaining why trade-offs are negotiated in the particular ways they are, we should not side-step the issue that educational and economic institutions are themselves socially constructed and coevolving entities, not wholly exogenous constraints on reproduction.

### (b) The meaning of education

Education is usually conceptualized as an economic indicator or as a proxy for embodied capital [10,12,73]. But education also exposes individuals to new ideas via other individuals, mass media and other sources of information, which effectively changes the entire landscape of options available to reproducing women. It potentially improves the fidelity and quality of the information received, reducing uncertainty in decision-making. Relatively little is known about how exactly education affects fertility, and the causal pathways will be different in different contexts [85]. This ambiguity is compounded at higher levels of aggregation, where education can proxy anything from the spatial distribution of economic development to higher rates of horizontal transmission. It is not enough to know that education correlates negatively with fertility at both a micro and a macro level. To generate causal hypotheses of fertility decline, we also need to know how its effects are determined, why humans have decided to value education as a social as well as an economic good, and how, when measured at different levels of aggregation, education influences all members of a population, not just the educated ones [15].

### (c) Prestige and competition

Prestige- or success-biases are entirely compatible with an approach to fertility decline that emphasizes inter-individual competition for parental investment payoffs. If individuals compete to obtain wealth or status, and these have fitness-relevant outcomes (which they have had for most of human history and in most pre-transition societies), then the behaviour of wealthy, high-quality parents is a useful cue to the benefits of reproductive control. It is therefore difficult to say whether the imitation of successful individuals is an indirect bias, in the sense that individuals copy other behaviours and end up with low fertility as a by-product, or a direct bias, where individuals adopt low fertility strategically. This makes it hard to distinguish them as alternative causal pathways.

### (d) Group-level effects

CE processes are multi-level phenomena. Yet, it is hard to know whether differences between particular levels of aggregation represent ‘cultural’ or ‘ecological’ determinants, and they can often be interpreted both ways. Are group-level effects on fertility indicative of cultural processes or aggregations of individual decisions in relation to some unobserved variable? Identification, selection and omitted variable problems are well known in economics and sociology [86] and are especially difficult to address when cultural and economic changes are correlated, as during demographic transitions. Theoretical refinements on the causal mechanisms of fertility decline as well as inventive data collection and analysis strategies will be needed to continue disentangling these effects.

Of course, the appropriate definition of a ‘group’ is not obvious, and no one social grouping (ego-networks, households, kinship groups, villages, social strata, ethnic, linguistic or religious groups) will be appropriate for all domains of social behaviour. Research focusing on differences and similarities between predictors at different levels of social hierarchies [28,87] will be increasingly important for such development.

### (e) Over-simplified assumptions?

Theoretical models of fertility decline usually assume undifferentiated individuals with equal opportunities to access information, perfect sampling of available cultural learning models and freedom to enact their reproductive preferences. CE models do not often include individual resource constraints on reproductive options, a hallmark of HBE, even though diffusion dynamics are sensitive to individual wealth or income heterogeneity [88], population sub-structure [89] and task structure [90].

CE models should also be tempered by the understanding that kin regularly have reproductive conflicts of interest and may wield significant power over reproductive-aged women. Dichotomies between kin and non-kin, implying broadly pro-natal outcomes of kin interactions, neglect the empirical evidence that kin effects on fertility are highly varied [91]. On the other hand, theorizing about kin conflict in HBE could be expanded to include how individuals negotiate the normative expectations of their kinship groups. CE dynamics may lead individuals to reject familial norms encouraging higher fertility, generating intergenerational conflict through alternative causal pathways to those that are typically considered in HBE.

CE models typically require that high-status individuals have lower fertility in order to obtain their results [21,58–60,68]. This one simplifying assumption begs the question: why do high-status individuals reduce fertility in the first place? Given the aspect of fertility decline being studied here (i.e. spread rather than origins), this assumption seems justified, and we should not expect all models to address all phases of the transition. However, CE should expand its focus to also address these origin-type questions. In principle, social learning can drive traits to fixation both when they are rare (via novelty-biases) and once they exist at an appreciable frequency, but we need to know how these preferences get to those frequencies and if we can distinguish them from asocial learning strategies.

### (f) Contraceptive use and uptake

Contraceptive use could represent strategic parental investment in the number or quality of children. But it could also represent a *dis*investment in reproduction in favour of other aims; self-fulfillment via education, work or some other measure of cultural success. For HBE, decisions to postpone, space or stop reproduction are sensitively tuned to environmental and social cues indicating optimal behaviour in a particular context, including cues of fecundability, mortality and resource availability throughout the life-course [4,92] and the support or disapproval of other kin [93,94]. Contraceptive use can be consistent with both high and low fertility, and users often have higher fertility than non-users in sub-Saharan Africa [93,95]. The focus on examining how frequencies of contraceptive behaviour change in CE research does not address the varying ways that contraceptives are used. CE also makes the problematic assumption that contraceptive use is synonymous with low-fertility preferences. This exposes a critical difference between contraceptive use as a proxy for low-fertility norms, subject to reproductive costs and benefits, and the type of method (or cultural variant) that an individual uses, which may diffuse entirely separately and through different transmission channels [28]. More broadly, the almost exclusive focus on the diffusion of ‘modern’ methods in both sub-fields obscures the fact that (i) all populations have probably tried to control their reproduction in some way [96], and (ii) natural methods such as *coitus interruptus* have been and remain critical in many fertility declines [28,97].

HBE has been more successful in accounting for the context-specific way that contraceptives are used, but less successful in explaining contraceptive diffusion, with network-based studies equivocal on the importance of social learning [13,28,93]. This raises yet another interpretive issue: should a lack of contraceptive clustering, in populations with very few users, be interpreted as evidence that social learning is unimportant? Or is an alternative interpretation that strong conformity to pre-existing traditional norms drives these results, especially given evidence that contraceptive information is widely available [13,93]?

## 5. Directions for future research

These conceptual overlaps suggest that we need to define more parts of the ‘system’ of fertility decline to articulate the added value of different approaches. This section outlines some suggestions for conceptual development, building on the foregoing literature.

### (a) Origins, spread and maintenance

Distinguishing between the origins, spread and maintenance of low fertility might be useful in defining the contours of different theoretical and empirical research programmes, highlighting the kinds of assumptions that models should make, and the different scales and processes that might matter. Doing so clarifies why most empirical work in HBE, focused primarily on the origins of low fertility, has been carried out in populations in the early stages of demographic transition [9,13,93,98] and perhaps also why evidence for social transmission has been hard to find. Similar work in populations at later stages of the transition has found strong effects consistent with cultural transmission models [15,28], further suggesting that predictors, but also processes, will differ depending on the context.

### (b) Multi-level selection

The potential role of multi-level or cultural group selection [63] has not yet been discussed with respect to fertility decline. That is, while maladaptive at the individual level, fertility decline may well be adaptive at (multiple) levels beyond the individual. When groups compete, selection can lead some groups to grow while others shrink (or even disappear). Members of the more successful group might have better survival and/or higher reproduction rates, individuals might selectively migrate to more attractive groups where they perceive life to be better [64], or out-group individuals might adopt characteristics from a group they regard as more successful. Group-level interactions are needed for a complete specification of the conditions favouring fertility decline because they may help generate new hypotheses, both about the evolution of educational and economic institutions, and the way these change the costs and benefits of reproduction in modernizing populations.

Historical fertility declines started in the wealthier sub-strata of technologically advanced populations during the transition from Malthusian stagnation to rapid economic growth, and are associated with profound social and economic changes brought about by the Industrial Revolution. Feedback between population density and technological innovation is thought to have then created a niche for education, to sustain subsequent economic growth [99]. An important difference between pre- and post-industrialized societies is the extent to which our populations are interconnected through labour and migration transfers, innovation and capital, and increasing interdependence in international trade and supply networks [66]. While migrants have been shown to rapidly adapt aspects of their value-systems within a single generation [100], whether this extends to reproductive norms requires further study [101]. Certainly, low fertility has been shown to help states become more wealthy, interconnected and market-oriented [102], generating higher *per capita* consumption through human capital accumulation. With increasing dependence on technology and innovation, countries sharing international research and development (R&D) and bilateral foreign direct investment are more economically productive than those that do not cooperate in this way [103,104]. Technologically advanced countries appear to interactively downregulate each other's fertility rates through competition and cooperation for increased economic productivity [66,105], and international trade also dampens fertility rates [106,107]. Fertility reductions cause temporary rises in the rate of economic growth via changing age structures and increasing the relative size of the labour force, a phenomenon known as the ‘demographic dividend’ [73,108].

Such population-level competition and cooperation may create selective pressure for market-oriented skills and investment in embodied capital. They can also benefit all individuals in a group, not just those who reduce fertility, for example by reducing mortality and increasing lifespans (24 years have been gained over the past century [102]) as populations develop better infrastructure [109]. Cultural or economic institutions, such as gender norms restricting women's employment, or structural biases in development spending, could cause groups to succeed or fail in this kind of intergroup competition. Cross-cultural differences in ‘tightness’ and ‘looseness’—for example, the acceptability of deviations from existing norms, the caution with which new norms are received and the openness of mass media and information flows—could also influence cultural transmission dynamics both within and between countries [110]. Economically or culturally successful countries may be able to spread their ideals more effectively than less successful ones, through ‘soft power’ or other means—indeed a prominent hypothesis in the demography literature is that fertility decline follows the spread of Western values [111]. Ultimately, the question is whether the benefits accrued from these higher-level dynamics outweigh the fitness costs in terms of individual fertility reduction. Multi-level research should be able to address these questions.

### (c) How does ‘structure’ affect fertility decline?

Some studies have started to address how demographic and social structure complicates inferences about the mechanisms of fertility decline. HBEs have recently begun to focus on how competition within, rather than between social strata affects reproduction [43,44,84], with some suggesting that fertility decline may be an example of Simpson's Paradox, where an overall negative relationship between wealth/status and fertility actually masks multiple stratified positive relationships [44]. CE approaches can provide additional insight into these questions. For example, we could conceptualize social strata as different groups with essentially different cultural norms. Imitating the reproductive behaviour of high-status individuals could then be regarded as a form of migration to a different group.

CE models have focused on how population-structure, age-structure and network composition affect the dynamics of cultural transmission [56,60,61]. Future work should try to combine these outlooks. Indeed, we need to know more about how cultural sub-structures, such as ethno-linguistic or religious groups, alter both information transmission dynamics and the costs and benefits of fertility decline in multicultural populations. In particular, cultural ‘outliers’ such as old order Anabaptists living in the US provide fascinating case studies where cultural norms effectively block reproductive change while allowing the selective use of economic innovations from outside the cultural group.

### (d) Recent ‘bounce back’ in fertility rates

How do the different evolutionary schools of thought interpret the recent ‘bounce-back’ in fertility rates in the most developed countries of the world [112]? One demographer has speculated that richer countries may end up with higher fertility than poorer ones in the future [113]. While many demographers are sceptical about a return to high fertility, since short-term baby booms in the recent past have not dampened the general trend towards low fertility, assuming that this will be a long-term equilibrium state without a strong theoretical grounding may be dubious [114]. HBEs might interpret bounce-back as a sign that the adaptive lag in human responses to our radically altered ecological niche is coming to an end, or that, as more people become wealthy and educated, the marginal advantages to investment in quality over quantity are declining, leading to relaxed reproductive competition and to higher fertility. Perhaps natural selection is shaping decision-making psychologies right now to explicitly value reproduction over status striving. Cognitive and other psychological research on this topic is sorely needed. Perhaps we will soon find the elusive evidence of a long-term fitness benefit, since lineages that reduced fertility most dramatically may gain a fitness advantage in the future as resources become scarcer and more unequally distributed [48,54]. CEs could argue that the relative stability of developed economies increases the benefits to asocial over social learning, or that social learning is now more accurately tracking environmental cues, either of which could de-emphasize horizontal transmission and potentially increase fertility rates. That richer countries may eventually end up with higher fertility than poorer countries also raises the possibility that multi-level selection is important. In the hypothetical future where global fertility has converged at or below replacement levels, populations with relatively younger age structures, owing to marginally higher fertility, may better compete internationally within global trade and communication networks.

## 6. Conclusion

Fertility decline is a difficult moving target, because no two declines are the same and because behaviour change involves multi-level processes and often, feedback. A comprehensive evolutionary approach to fertility decline must incorporate insights from CE theory if it is to fully understand this transition process. Doing so requires a greater degree of multi-level thinking and a deeper understanding of the ‘proximate’ mechanisms that are often seen as a secondary concern. Mechanistic causal models are needed because theories that are functionally equivalent are not necessarily causally equivalent [41]. A focus on the ultimate function of behaviour has undoubtedly been successful in illuminating a wide array of human behaviours in less- and more-developed environments and in generating new hypotheses. But a clearer understanding of how individuals go about the business of optimizing fitness, and whether optimization is robust to information cascades and multi-level cultural dynamics, is needed. A conceptual division between the origin, spread and maintenance of low fertility may be useful in tackling these problems, without asserting causal primacy to one level of analysis over another. Multi-level cultural selection is an open topic of debate and refinement [38,41,63], and researchers of fertility decline are well placed to contribute to these broader theoretical developments, not least because reproduction is the primary mechanism of biological evolution. Combined with attention to overlapping debates in demography, a synthesis of evolutionary frameworks will provide a better understanding than will a focus on one framework alone.
